# Pigment loss and pseudo-albinism in Birdshot chorioretinitis

**DOI:** 10.1038/s41433-026-04335-1

**Published:** 2026-03-05

**Authors:** Aliénor Vienne-Jumeau, Caroline Memmi, Souhila Kecili, Jordan Loeliger, Jennifer E. Thorne, Dominique Monnet, Antoine P. Brézin

**Affiliations:** 1 Ophtalmopôle, Cochin Hospital, Assistance Publique-Hôpitaux de Paris, Université Paris Cité, Paris, France; 2 CHNO des Quinze-Vingts, INSERM-DGOS CIC 1423, 28 rue de Charenton, Paris, France; 3 Department of Ophthalmology, University Hospital Basel, Basel, Switzerland; 4 Division of Ocular Immunology and Uveitis, Wilmer Eye Institute, Johns Hopkins University School of Medicine, Baltimore, MD USA; 5 Department of Epidemiology, Johns Hopkins Bloomberg School of Public Health, Baltimore, MD USA

**Keywords:** Outcomes research, Uveal diseases

## Abstract

**Objectives:**

To describe a rare phenotype of birdshot chorioretinitis (BSCR) characterised by diffuse retinal depigmentation and to evaluate its relationship with disease chronicity and visual outcomes.

**Methods:**

This observational study included patients from the CO-BIRD cohort with ultra-widefield fundus imaging. Retinal depigmentation was graded using the albinism scale by Kruijt et al. (grades 0–3). Eyes with a grade ≥ 2 in at least one image were included. Disease activity and severity were assessed using best-corrected visual acuity (BCVA), vision-related quality of life (VFQ-25), visual field testing and multimodal imaging.

**Results:**

Among 1016 eyes (508 patients), 52 eyes (26 patients, 5.1%) had depigmentation grade ≥ 2; 22 eyes (2.2%) reached grade 3. Fifteen years after symptom onset, BCVA worse than 0.7 logMAR occurred in 42.1% of grade 3 versus 5.0% of grade 2 eyes (*p* = 0.014). VFQ-25 scores were similarly reduced in both groups. Grade 3 was associated with a lower risk of optic disc oedema (HR = 0.38; *p* = 0.028) and a non-significant reduction in macular oedema risk. Choroidal neovascularisation occurred only in grade 3 (40.9%). Grade 3 was linked to worse BCVA (*p* = 0.012), higher pattern standard deviation (*p* = 0.011), and a history of active disease. Compared with previously published CO-BIRD data, grade 3 showed faster BCVA decline (*p* = 0.044) and greater cumulative loss (*p* = 0.001).

**Conclusion:**

Diffuse retinal depigmentation resembling albinism is an uncommon but severe expression of BSCR, indicative of chronic disease and associated with poorer visual outcomes.

## Introduction

Birdshot chorioretinitis (BSCR) is a rare, chronic form of posterior uveitis that primarily affects individuals of middle age and European descent [1]. It is considered an autoimmune condition, strongly associated with HLA-A29 and linked to variants in ERAP1 and ERAP2, suggesting a role for altered antigen processing and presentation [2]. While traditionally described as a well-defined clinical entity, BSCR exhibits marked heterogeneity in its presentation, disease course, and response to treatment.

A key feature of BSCR is the presence of multiple, cream-coloured, ovoid choroidal lesions radiating from the optic disc, which serve as a cornerstone for diagnosis [3]. However, in clinical practice, these lesions are not always easily visible—particularly in early or atypical cases where pigmentation is subtle, the media is hazy, or lesions are confluent. This can delay diagnosis and treatment initiation, contributing to avoidable visual morbidity [4]. In such cases, indocyanine green angiography may be critical in revealing occult hypofluorescent lesions, improving diagnostic sensitivity [5].

To date, most of the literature has focused on the characterisation of birdshot lesions themselves, including their topography, pigmentation, and progression over time [1, 6]. In contrast, relatively little attention has been paid to the phenomenon of retinal depigmentation, particularly when diffuse or confluent. Depigmentation is typically interpreted as a late or residual finding, reflecting past damage rather than ongoing disease activity, and its clinical relevance remains poorly defined. Importantly, not all patients develop widespread depigmentation. While confluent plaques—typically extending from the inferonasal retina—are common, some patients exhibit only focal changes, while others develop diffuse chorioretinal atrophy. The clinical significance of these depigmentation patterns, particularly in relation to visual function, remains poorly understood.

In this study, we report a subgroup of patients with BSCR who exhibited diffuse retinal depigmentation and investigate the functional implications of this presentation. To characterise the extent of depigmentation in a reproducible manner, we applied a grading scale (ranging from 0 to 3) previously developed by Kruijt et al. in the context of ocular albinism, which assesses choroidal transparency as a proxy for fundus pigmentation [7]. In this system, grades 0 and 1 correspond to a normally pigmented fundus and depigmentation limited to the (mid)periphery, respectively. Grade 2 is defined by the visibility of choroidal vessels in the posterior pole but sparing the macular region, while grade 3 denotes choroidal vessel visibility extending into the macula itself (modified from Summers et al. [8]). Although initially designed for congenital hypopigmentation syndromes, this scale appears applicable for phenotyping BSCR patients with retinal depigmentation, offering a structured approach to evaluate its potential prognostic value. By comparing visual outcomes across depigmentation grades, we aim to assess whether diffuse retinal depigmentation is associated with more severe or accelerated visual decline, and whether the extent of depigmentation may serve as a prognostic marker in BSCR.

## Materials and methods

### Study design

This study draws on data from the CO-BIRD cohort (COhort of patients with BIRDshot chorioretinitis—ClinicalTrials.gov Identifier: NCT05153057), conducted at a single centre, Hôpital Cochin in Paris, France. Initiated in 2002 and still ongoing, this prospective cohort includes patients undergoing a standardised annual evaluation. Details of the data collection procedures have been described previously [4]. The diagnosis of the disease was established according to the criteria from an International Consensus Conference and subsequently confirmed by the Standardization of Uveitis Nomenclature (SUN) group [3].

### Patients

Patients were included from the CO-BIRD cohort if they had at least one eligible Optos® ultra-widefield image. From this cohort, we identified a group of patients exhibiting retinal depigmentation graded ≥2 in at least one ultra-widefield image during follow-up, based on the albinism grading system proposed by Kruijt et al. [7] and adapted from Summers et al. [8]. This scale defines four grades: grade 0 corresponds to normal pigmentation; grade 1 indicates visibility of choroidal vessels in the (mid)periphery; grade 2 indicates visibility of choroidal vessels in the posterior pole but not in the macular region; and grade 3 indicates visibility of choroidal vessels within the macular region.

### Data collection

For these patients, all available prior and subsequent fundus images—ultra-widefield when available, or four-field retinographies for earlier dates before the adoption of ultra-widefield imaging in 2018—were reviewed, and Kruijt grades were assigned at each visit. Additional data collected at each visit included best-corrected visual acuity (BCVA), axial length (AL), slit-lamp examination with grading of anterior chamber and vitreous cells, spectral-domain optical coherence tomography (SD-OCT) scans of the macula and optic nerve, fluorescein angiography (FA) to assess venous vasculitis, vision-related quality of life assessed using the National Eye Institute Visual Function Questionnaire—25 item version (VFQ-25), and visual field-testing using Humphrey automated perimetry when feasible. In patients with advanced visual field defects for whom Humphrey perimetry could not be performed, Goldmann perimetry was used instead, and the mean deviation (MD) was assigned a value of −30 dB for study purposes, as previously reported [9]. Disease duration was recorded based on both the date of diagnosis and the reported onset of visual symptoms. Transient vision loss due to advanced cataract—defined as a decline of >0.2 logMAR (worse than 20/32 Snellen) on ≤2 consecutive visits, followed by subsequent cataract extraction—was not considered in the analysis.

### Statistical analysis

Descriptive statistics were used to summarise the study population. Categorical variables were compared using Fisher’s exact test, and continuous variables were assessed for normality with the Shapiro–Wilk test. Normally distributed variables are reported as mean ± SD, and non-normally distributed variables as median (IQR). Associations between depigmentation grade and functional outcomes (BCVA, MD, PSD) were analysed using Generalized Estimating Equations (GEE) to account for correlation between eyes of the same patient. Univariate models included Kruijt group (3 vs <3), time to evaluation, time to diagnosis, age at diagnosis, and history of MO, ODO, or CNV. Variables with *p* < 0.1 were entered into multivariable models. Longitudinal changes in BCVA, MD, and PSD by the Kruijt group, and compared with the CO-BIRD cohort,⁹ were assessed using linear mixed models (LMM) for slopes and area between curves (ABC). Time-to-event endpoints (MO, ODO, CNV) were analysed with left-truncated, right-censored Kaplan–Meier curves, log-rank tests, and Cox models. Vision-related quality of life was evaluated using the National Eye Institute Visual Function Questionnaire—25-item version (VFQ-25). Correlations between VFQ-25 scores and BCVA of the better eye at the first available VFQ assessment were tested with Spearman’s rank correlation. The proportion of patients with subscale or composite scores < 50 at 15 ± 5 years after symptom onset was compared between Kruijt grades using two-sample proportion tests. Because we tested a prespecified set of outcomes (no item-level analyses), no multiplicity correction was applied for this analysis, and *p*-values are reported as two-sided and exploratory. All analyses were conducted in R (version 2024.09.1).

### Ethics statement

This study involves human participants, and the study was approved by the ’Comité Consultatif de Protection des Personnes dans la Recherche Biomédicale’ (ethics approval id 00011558) and maintained adherence to the Declaration of Helsinki for research involving human subjects. Informed consent was obtained from all participants before study enrolment. Participants gave informed consent to participate in the study before taking part.

## Results

### Patients’ characteristics

**Fig. 1:**
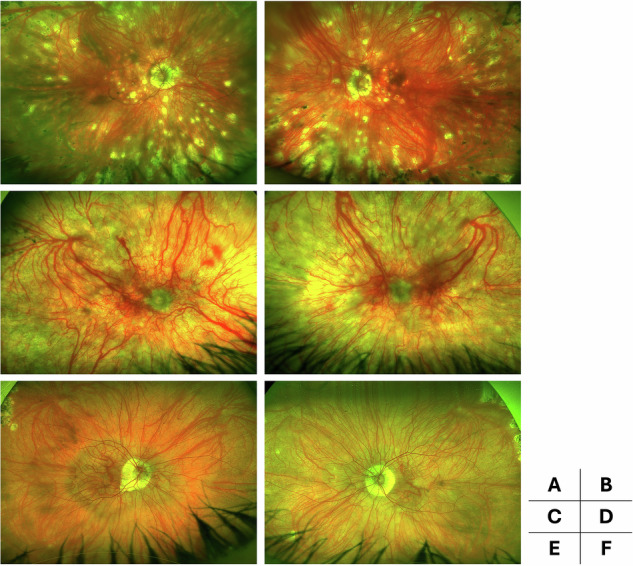
Representative cases of widefield fundus imaging (Optos, Optomap®) in three patients with Birdshot chorioretinitis and Kruijt grade 3 depigmentation. Patient 1 shows numerous atrophic, occasionally pigmented spots with coalescence in the posterior pole (**A**, **B**), whereas the other two patients exhibit fewer spots but diffuse choroidal depigmentation throughout the posterior segment (**C**–**F**).

Among the 508 patients (1016 eyes) in the CO-BIRD cohort with available wide-field imaging, 52 eyes of 26 patients (5.1%) met the inclusion criteria. Twenty-two eyes (2.2%) reached the highest level of fundus depigmentation (Kruijt grade 3) and 29 (2.9%) reached a maximum of grade 2 during follow-up. Figure 1 illustrates three examples of grade 3 depigmentation. Twenty-four of the 26 patients (92%) were female. The mean age at diagnosis was 56.4  ±  11.5 years, and the mean age at first evaluation in our centre was 59.4  ±  12.3 years. The mean diagnostic delay was 3.0  ±  5.0 years, and the mean disease duration at first evaluation was 6.2  ±  6.1 years. The VFQ-25 composite score at first evaluation was 54.8 ± 30.6. The mean follow-up duration was 11.2  ±  6.3 years. At the first evaluation, the mean BCVA was 0.27 ± 0.43 LogMAR (20/37 ± 20/53 Snellen). Two eyes (3.8%) had CNV at baseline, and seven eyes (13.5%) developed CNV during follow-up. Macular oedema (MO) was present in 11 eyes (21.1%) at baseline and occurred in 19 eyes (36.5%) during follow-up. Optic disc oedema (ODO) was observed in 23 eyes (44.2%) at baseline and in 12 eyes (23.1%) during follow-up. The detailed demographic and clinical characteristics are summarised in Table 1.

**Table 1:** Data of included patients.

*Patient-level variables*	26 patients
Gender, Female, *n* (%)	24 (92.0%)
Age at diagnosis (years), mean ± SD	56.4 ± 11.5
Age at first evaluation (years), mean ± SD	59.4 ± 12.3
Diagnostic delay (years), mean ± SD	3.0 ± 5.0
NEI VFQ-25 composite score at first evaluation, mean ± SD	54.8 ± 30.6
Disease duration at first evaluation (years), mean ± SD	6.2 ± 6.1
Follow-up duration (years), mean ± SD	11.2 ± 6.3
**Eye-level variables**	**52 eyes**
Axial length (mm), mean ± SD	23.9 ± 1. 5
Kruijt score, median [IQR]	
At the first evaluation	2 [1–2]
At the last evaluation	2 [2–3]
BCVA (LogMAR), mean ± SD	
At the first evaluation	0.27 ± 0.43
At the last evaluation	0.51 ± 0.8
CNV, *n* (%)	
At the first evaluation	2 (3.8%)
During follow-up	7 (13.5%)
MO, *n* (%)	
At the first evaluation	11 (21.1%)
During follow-up	19 (36.5%)
ODO, *n* (%)	
At the first evaluation	23 (44.2%)
During follow-up	12 (23.1%)

*BCVA* best corrected visual acuity, *CNV* choroidal neovascularisation, *MO* macular oedema, *ODO* optic disc oedema, *NEI VFQ-25* National Eye Institute Visual Function Questionnaire—25 item version.

### Evolution of fundus depigmentation and association with BCVA loss

Supplementary Fig. 1 presents a lasagna plot of Kruijt fundus depigmentation grades over time for each eye, revealing a median interval of 9.0 years (IQR 4.0–12.0) from first symptoms to reach grade 2 and 13.7 years (IQR 6.3–21.7) to reach grade 3 (log-rank *p* = 0.006). Although some eyes progressed to grade 3 in less than 6 months and others remained at lower grades for over 20 years, by 10 years all eyes had developed at least grade 2 and none ever regressed. Supplementary Fig. 2 illustrates the corresponding heterogeneity in BCVA (logMAR) trajectories over the same period. Finally, at 15 ± 5 years after symptom onset, 42.1% of eyes with a baseline Kruijt grade 3 had a BCVA worse than 0.7 logMAR (equivalent to worse than 20/100 Snellen), compared to only 5.0% of eyes with grade 2 at baseline. This difference remained significant after accounting for within-patient clustering in a GEE model (Wald *p* = 0.014) (Supplementary Fig. 3). The Composite score of the VFQ-25 did not correlate with BCVA of the better eye (Spearman, *R* = –0.49, *p* = 0.18). Among the subscales, Ocular Pain (*R* = –0.83, *p* = 0.003), General Vision (*R* = –0.77, *p* = 0.010), Driving (*R* = –0.78, *p* = 0.024) correlated significantly with BCVA, whereas General Health, Near Activities, Distance Activities, Vision Social, Vision Mental Health, Vision Role, Vision Dependency, Color Vision, Peripheral Vision did not (all *p* ≥ 0.05). At 15 ± 5 years after symptom onset, the proportion of patients with VFQ-25 subscale scores below 50 was comparable between Kruijt grades 2 and 3 across all domains, with values ranging from 25.0% to 91.7% in Kruijt 2 and from 42.9% to 71.4% in Kruijt 3. The composite score was < 50 in 58.3% of Kruijt 2 and 57.1% of Kruijt 3 patients (*p* = 0.96). Differences were not statistically significant for any subscale (all *p* ≥ 0.16).

### Risk of macular or papillary events

**Fig. 2:**
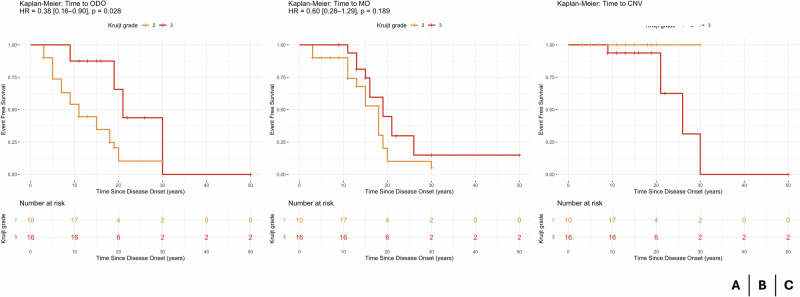
Kaplan–Meier survival curves for complications. Kaplan–Meier survival curves showing time to first occurrence of **A** optic disc oedema (ODO), **B** macular oedema (MO), and **C** choroidal neovascularisation (CNV), stratified by Kruijt grade. Log-rank *p*-values and hazard ratios (HRs) are indicated for MO and ODO and were not computed for CNV (no event in group Kruijt 2).

In a delayed-entry Kaplan–Meier analysis, eyes with Kruijt grade 3 depigmentation had a significantly lower risk of ODO than those with grade 2 (hazard ratio [HR] 0.38, 95% confidence interval [CI] 0.16–0.90; *p* = 0.028). The median ODO-free survival was 21.0 years for grade 3 versus 11.0 years for grade 2 eyes. For MO, grade 3 eyes likewise exhibited a reduced—but non-significant—risk compared with grade 2 (HR 0.60, 95% CI 0.28–1.29; *p* = 0.189), with median MO-free survival of 19.0 years versus 18.0 years. By contrast, CNV developed in 40.9% of grade 3 eyes but in none of the grade 2 eyes; among affected grade 3 eyes, the median time to CNV onset was 26.0 years (IQR 21.0–30.0). All CNV events occurred exclusively in grade 3 eyes and late in follow-up, precluding direct time-to-event comparisons between grades. (Fig. 2)

### Disease activity and treatment

Supplementary Table 1 summarises disease activity and treatment characteristics. A history of venous vasculitis was present in 17/26 (65%) patients, 1+ anterior chamber cells (Tyndall) in 2/26 (8%), and +1 vitreous cells (vitritis) in 20/26 (77%). Systemic corticosteroids were used in 9/26 (35%) patients, sub-Tenon corticosteroid injections in 15/26 (58%), and intravitreal corticosteroids in 11/26 (42%). Additionally, 17/26 (65%) patients received systemic immunosuppression.

When stratified by initial depigmentation grade, systemic corticosteroid and immunosuppressive exposure did not differ significantly between patients who already had Kruijt grade 3 at first presentation (0 [0%] and 2 [40.0%], respectively) and those with initial Kruijt < 3 (9 [42.9%] and 15 [71.4%], respectively; *p* = 0.13 and *p* = 0.30, Fisher’s exact test). Similarly, at the last follow-up, systemic corticosteroid and immunosuppressive exposure did not differ significantly between patients who reached grade 2 (6 [42.9%] corticosteroids; 10 [71.4%] immunosuppression) and those who reached grade 3 (3 [25.0%] corticosteroids; 7 [58.3%] immunosuppression; *p* = 0.43 and *p* = 0.68, respectively).

### Univariate analysis of factors associated with functional outcomes

In univariate GEE models (Table 2), Kruijt grade 3 was significantly associated with worse BCVA (LogMAR) (*β* = 0.56, SE = 0.22; *p* = 0.012), a tendency to greater MD loss (*β* = –3.99 dB, SE = 2.21; *p* = 0.071, trend), and higher PSD (*β* = 1.12 dB, SE = 0.44; *p* = 0.011). Each additional five-year delay from first symptoms to evaluation worsened BCVA (*β* = 0.17, SE = 0.05; *p* = 0.0002), MD (*β* = –1.51 dB, SE = 0.37; *p* < .001) and PSD (*β* = 0.56 dB, SE = 0.16; *p* = 0.0004). By contrast, delay to diagnosis (per five years) did not reach significance for any outcome. History of MO showed trends toward worse BCVA and MD (*p* = 0.07), while history of ODO and of CNV were each significantly linked to poorer BCVA (ODO: *β* = 0.40, SE = 0.19, *p* = 0.036; CNV: *β* = 1.15, SE = 0.32, *p* = 0.0003) and history of CNV also to greater MD loss (*β* = –5.06 dB, SE = 2.19, *p* = 0.021).

**Table 2:** Univariate GEE analysis of factors associated with functional outcomes (BCVA, MD, PSD) among the 26 included patients.

		Estimate	SE	*p*-value
BCVA (LogMAR)	**Kruijt Group 3**	0.56	0.22	**0.012**
	Time from First Symptoms to Evaluation (years)	0.17	0.05	**0.0002**
	Time from First Symptoms to Diagnosis (years)	0.01	0.01	0.35
	Age at Diagnosis	0.01	0.01	0.14
	History of MO	0.28	0.16	0.073
	History of ODO	0.40	0.19	**0.036**
	History of CNV	1.15	0.32	**0.0003**
MD (dB)	Kruijt Group 3	–3.99	2.21	0.071
	Time from First Symptoms to Evaluation (years)	–1.51	0.37	**<.0001**
	Time from First Symptoms to Diagnosis (years)	–0.19	0.17	0.28
	Age at Diagnosis	–0.19	0.12	0.12
	History of MO	–3.07	1.71	0.073
	History of ODO	–0.84	0.87	0.34
	History of CNV	–5.06	2.19	**0.021**
PSD (dB)	Kruijt Group 3	1.12	0.44	**0.011**
	Time from First Symptoms to Evaluation (years)	0.56	0.16	**0.0004**
	Time from First Symptoms to Diagnosis (years)	0.01	0.03	0.63
	Age at Diagnosis	0.04	0.05	0.36
	History of MO	1.03	0.51	**0.042**
	History of ODO	0.52	0.32	0.10
	History of CNV	0.65	0.81	0.42

Estimates are regression coefficients from exchangeable correlation generalized estimating equations (GEE) models, with standard error (SE) and *p*-value for each predictor. Time from First Symptoms is scaled per 5-year increase. Kruijt group compares patients with Kruijt score 3 vs <3.

*BCVA* best corrected visual acuity (LogMAR), *MD* mean deviation (dB), *PSD* pattern standard deviation (dB), *SE* standard error, *MO* macular oedema, *ODO* optic disc oedema, *CNV* choroidal neovascularisation.

Bold values indicate statistical significance *p* < 0.05.

### Multivariable analysis of independent predictors

**Fig. 3:**
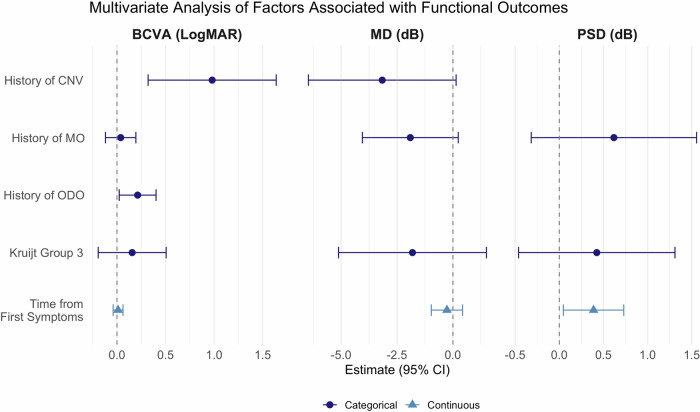
Multivariate analysis of factors associated with long-term visual and functional outcomes. Forest plots show the effect sizes (with 95% confidence intervals) for each variable on best-corrected visual acuity (BCVA, logMAR), mean deviation (MD, dB), and pattern standard deviation (PSD, dB) at final follow-up. Filled circles represent categorical variables, while filled triangles represent continuous variables. Kruijt grade 3, longer time from first symptoms to evaluation, and history of macular oedema (MO), optic disc oedema (ODO), or choroidal neovascularisation (CNV) were assessed as predictors of poorer outcomes.

When all covariates with univariate *p* < 0.10 were entered simultaneously, only history of ODO (*β* = 0.21, SE = 0.10; *p* = 0.028) and CNV (β = 0.98, SE = 0.34; *p* = 0.004) remained independently associated with worse BCVA. No factor achieved statistical significance for MD after adjustment, though delay to evaluation trended toward association (*β* = –0.80 dB per 5 years, SE = 0.45; *p* = 0.077). For PSD, time from first symptoms to evaluation remained an independent predictor (*β* = 0.39 dB per 5 years, SE = 0.17; *p* = 0.027); all other variables lost significance in the multivariable models. (Supplementary Table 2) The forest plot (Fig. 3) highlights the relative magnitude and precision of these adjusted effects across outcomes.

### Comparison of visual function trends between Kruijt groups at the last evaluation

The evolution of BCVA in our cohort was compared with normative data from the CO-BIRD cohort previously published. (Fig. 3 and Supplementary Table 3) In a head‑to‑head comparison against published five‑year norms [9], BCVA in Kruijt grade 2 eyes progressed at a significantly different linear rate (slope-difference *z* = 2.14, Trend *p* = 0.016. However, the overall trajectory remained tightly clustered around the reference (area‑between‑curves permutation *p* = 0.99), indicating minimal cumulative divergence. Kruijt grade 3 eyes showed a BCVA trajectory that differed significantly in both changed at a rate distinct from the whole CO-BIRD cohort (Trend *p* = 0.044) and showed a highly significant cumulative difference (ABC *p* = 0.001) compared to previously published five-year data from the full CO-BIRD cohort. MD trajectories did not differ in slope from the published norms for either Kruijt grade (both Trend *p* > 0.05), but the cumulative area-between-curves was nonetheless significant (ABC *p* = 0.007), indicating that MD loss in our cohort diverges modestly but meaningfully from expected values over time. PSD closely mirrored the previously published cohort data in both rate of change and overall trajectory. (Supplementary Fig. 4)

## Discussion

We identified and characterised a subgroup of BSCR patients showing extensive retinal depigmentation, reminiscent of pseudo-albinism, which appears to represent an advanced stage or chronic expression of the disease. By stratifying patients according to the degree of depigmentation using the grading system proposed by Kruijt et al. for albinism [7], we observed distinct structural and functional trajectories. Although individual disease courses were heterogeneous, no patient in our cohort showed a reversal to a lower Kruijt grade over time. Importantly, most patients had received corticosteroid and/or systemic immunosuppressive therapy, reflecting ongoing or previously active disease. Advanced depigmentation (grade 3) was associated with a more rapid and pronounced decline in visual function compared to intermediate depigmentation (grade 2), suggesting that the extent of retinal depigmentation reflects a more aggressive and progressive disease course. When compared to published data on the whole CO-BIRD cohort [9], these patients also exhibited more severe and accelerated visual deterioration (BCVA and MD). Taken together, these findings suggest that both the presence and extent of diffuse retinal depigmentation—particularly when involving central areas such as the macula—may carry significant prognostic value in BSCR. The occurrence of choroidal neovascularisation (CNV) in patients with macula-involving depigmentation (grade 3) may further contribute to the greater decline in BCVA observed in this subgroup. Notably, CNV was more frequent in our grade 3 cohort (40.9%) compared to previously reported rates in the literature (11–13.5%) [10, 11].

The BCVA in the most advanced depigmentation group (grade 3) not only worsened more steeply than the rest of the CO-BIRD cohort but also drifted significantly away from the reference trajectory when viewed across all timepoints (trend *p* = 0.044; ABC *p* = 0.001). By contrast, the intermediate group (grade 2) showed an accelerated decline in slope (trend *p* = 0.016) that, despite its statistical significance, translated into minimal net deviation from the published curve (ABC *p* = 0.997)—their acuity values remained essentially within the anticipated band. When we examined visual field loss, the late-stage group again revealed a subtle but important pattern: although its rate of change did not differ from norms (trend *p* > 0.05), the cumulative loss over time was significant (ABC *p* = 0.007), uncovering a gradual divergence that the slope test alone would miss. Collectively, these results delineate a graded pattern of progression: eyes with moderate depigmentation exhibit an accelerated rate of decline that nonetheless remains aligned with normative trajectories, whereas those with severe depigmentation demonstrate both a faster decline and a significant cumulative deficit—particularly regarding visual acuity—as well as a progressively greater visual‑field loss over time. That said, the observed association between grade 3 depigmentation and functional decline should not be interpreted as conclusive evidence of causality. It remains uncertain whether depigmentation precedes, parallels, or follows functional loss. In this context, the finding that some patients with advanced depigmentation retain relatively preserved visual function (patients 1, 2, 3, 16 and 22 from Supplementary Fig. 2) is especially noteworthy and suggests that structural and functional changes may not always evolve in parallel.

While the pathophysiological significance of retinal depigmentation in BSCR remains uncertain, several hypotheses may be considered. Depigmentation could represent the cumulative consequence of longstanding, uncontrolled inflammation, leading to widespread loss of retinal pigment epithelium (RPE) integrity and choroidal hypopigmentation. Delayed diagnosis is a well-recognised challenge in BSCR, owing to its insidious onset and the absence of early pathognomonic signs. In theory, pseudo-albinism could result from the gradual coalescence of classic birdshot lesions over time. However, although some patients with grade 2 and grade 3 depigmentation did experience a prolonged delay between symptom onset and diagnosis, the median diagnostic delay in this group (3 years) was similar to that reported for the broader BSCR population [9].

Alternatively, depigmentation may be an active component of disease pathology—potentially indicating areas of intense immune-mediated injury or high lymphocytic infiltration in the choroid. In this framework, pseudo albinism may not merely be a late-stage epiphenomenon, but a surrogate marker of a high inflammatory burden. Notably, patients in grade 2 exhibited high rates of early ODO and MO, and those in grade 3 were prone to developing CNV during follow-up. One possibility is that grade 3 depigmentation reflects a more advanced stage of disease, with grade 2 representing an intermediate step along a continuum of progressive chorioretinal damage. The longer mean disease duration observed in grade 3 eyes—approximately 5 years more than in grade 2—supports this interpretation. In this later stage, inflammatory signs such as MO and ODO may become less frequent or less detectable, not because of a genuinely less inflammatory phenotype, but due to underlying tissue degeneration and retinal exhaustion. In this framework, the absence of acute inflammatory markers may reflect a shift toward a quiescent or atrophic phase of disease, rather than a true reduction in disease activity. However, the presence of overlapping disease durations and comparable ages in some patients across grades 2 and 3 argues against a strictly sequential model. Instead, these findings suggest that grades 2 and 3 may represent, at least in part, distinct phenotypic expressions within the broader BSCR spectrum, rather than consecutive stages of a single pathophysiological process. This temporal and phenotypic divergence may reflect different phases or immune mechanisms within the disease and warrants further investigation.

Parallels have been drawn with other inflammatory disorders such as Vogt–Koyanagi–Harada disease and melanoma-associated retinopathy, which are characterised by progressive choroidal depigmentation secondary to immune-mediated melanocyte destruction [12, 13]. In BSCR, a similar mechanism has been postulated. Histopathology demonstrates nodular infiltrates composed of T and B lymphocytes, along with myeloid lineage cells, within the choroid and ciliary body [14, 15]. Hassman et al. detected circulating anti-melanoma antibodies in five patients with BSCR, suggesting a possible cross-reactive immune response targeting melanocytic antigens [16]. However, unlike VKH and MAR, BSCR is marked by early retinal involvement, often presenting with ODO, cystoid MO, and optic disc pallor [17, 18]. This observation suggests that additional, retina-specific inflammatory mechanisms may be at play.

Several limitations of our study must be acknowledged. The primary limitation is the small sample size, which reflects the rarity of this extreme phenotype within the broader BSCR population. Additionally, different fundus imaging modalities were used over time—four-field retinographies prior to 2018 and ultra-widefield imaging thereafter—which may have introduced variability in the assessment and grading of chorioretinal lesions. In our longitudinal design, patients with longer follow-up durations contributed more data points, potentially introducing selection bias. The reported onset of visual symptoms may also be subject to recall bias; however, given that time intervals were grouped into five-year categories, we believe this provided a sufficient buffer to account for such imprecision. Various ocular comorbidities—such as cataract, epiretinal membrane formation, and glaucoma—may have influenced visual outcomes, although our methods attempted to limit these confounding factors. To minimise this source of confounding, we excluded visits during which cataract caused a transient and clinically significant decrease in BCVA, defined as a loss of >0.2 logMAR (worse than 20/32 Snellen) on no more than two consecutive visits, followed by recovery after cataract extraction. No patients in our cohort had late-stage glaucoma. In two with advanced visual field loss, Goldmann perimetry was used and an MD value of –30 dB was assigned, following CO-BIRD methodology to preserve comparability despite potential bias.

Overall, our findings suggest that pseudo-albinism may represent a severe, end-stage form of BSCR in which characteristic lesions become undetectable, challenging current diagnostic criteria. The underlying mechanisms remain unclear, and optimal strategies for prevention have yet to be defined.

## Summary

### What is known about this topic

- Diffuse retinal depigmentation—resembling pseudo albinism—has been described anecdotally as a late feature of birdshot chorioretinitis (BSCR)
- Its prevalence and impact on vision remain unquantified.

### What this study adds

- This study identifies and characterises a rare pseudo albinism phenotype in BSCR (Kruijt grades 2–3), present in 7% of eyes, and demonstrates that depigmentation can be reliably graded using an albinism based classification scale.
- Moderate depigmentation (grade 2) is associated with a significant decline in best-corrected visual acuity (BCVA), but severe depigmentation (grade 3) leads to a steeper decline and uniquely predisposes to choroidal neovascularisation.
- In univariate analyses, grade 3 eyes were significantly associated with worse BCVA and with greater visual field loss; in multivariate models, history of optic disc oedema and choroidal neovascularisation (CNV) remained independent predictors of poorer BCVA.

## Supplementary information

Supplementary Table 1

Supplementary Table 2

Supplementary Table 3

Supplementary Figure 1

Supplementary Figure 2

Supplementary Figure 3

Supplementary Figure 4

## Data Availability

The data supporting the findings of this study are available from the corresponding author upon reasonable request.
