# Supplementary material for: Pigment loss and pseudo-albinism in Birdshot chorioretinitis
Source: Eye (Lond). 2026 Mar 5;40(8):1162–8. doi: 10.1038/s41433-026-04335-1 (PMC13195155; doi:10.1038/s41433-026-04335-1)
Supplement: Supplementary file 1 — Supplementary Table 1 [file 41433_2026_4335_MOESM1_ESM.docx]

| Id | Kruijt at first visit | Kruijt at last visit | Gender | Age at first signs | Time between first signs and diagnosis (years) | ODO history | MO history | Venous vasculitis history | CNV history | Tyndall history | Hyalitis history | History of systemic CS | History of subtenon CS | History of intravitreal CS | IS | IS type |
| --- | --- | --- | --- | --- | --- | --- | --- | --- | --- | --- | --- | --- | --- | --- | --- | --- |
| Patient 1 | 2 / 2 | 3 / 3 | F | 36 | 1 | yes / yes | no / no | no / yes | yes / no | yes / yes | yes / yes | yes | no / no | yes / no | yes | MMF then ADM |
| Patient 10 | 2 / 2 | 3 / 3 | F | 72 | 3 | yes / yes | yes / yes | yes / yes | yes / yes | no / no | yes / yes | no | no / no | yes / no | no | no |
| Patient 11 | 2 / 2 | 3 / 3 | F | 49 | 5 | no / no | no / yes | no / no | no / no | no / no | no / yes | no | no / no | yes / no | yes | ADM then stop |
| Patient 12 | 2 / 2 | 2 / 2 | F | 48 | 4 | yes / yes | yes / yes | yes / yes | no / no | no / no | yes / yes | no | yes / no | no / no | yes | MMF then ADM then MMF + ADM |
| Patient 13 | 2 / 2 | 2 / 2 | F | 66 | 4 | yes / yes | yes / yes | yes / yes | no / no | no / no | no / no | no | no / yes | no / no | yes | MMF then stop |
| Patient 14 | 1 / 1 | 3 / 3 | F | 62 | 1 | yes / yes | yes / yes | yes / yes | yes / yes | no / no | yes / yes | no | no / no | yes / yes | no | no |
| Patient 15 | 2 / 2 | 2 / 2 | F | 64 | 1 | yes / yes | yes / no | yes / yes | no / no | no / no | yes / yes | no | no / yes | no / no | yes | MTX |
| Patient 16 | 3 / 3 | 3 / 3 | F | 38 | 3 | yes / yes | yes / yes | no / yes | no / no | no / no | yes / yes | no | no / yes | no / no | yes | MMF then stop |
| Patient 17 | 1 / 1 | 2 / 3 | F | 53 | 4 | yes / no | yes / yes | yes / yes | no / no | no / no | yes / yes | yes | no / no | no / no | yes | MMF |
| Patient 18 | 2 / 2 | 2 / 2 | F | 54 | 1 | yes / yes | yes / yes | yes / yes | no / no | no / no | yes / yes | no | no / yes | yes / yes | yes | MMF then ADM then AZT |
| Patient 19 | 1 / 1 | 2 / 2 | F | 59 | 1 | yes / yes | no / no | yes / yes | no / no | no / no | no / no | no | yes / yes | no / no | no | no |
| Patient 2 | 0 / 0 | 3 / 3 | M | 50 | 1 | no / no | no / no | no / no | no / no | no / no | yes / yes | no | no / no | no / no | yes | MMF then stop |
| Patient 20 | 1 / 2 | 2 / 2 | F | 50 | 0 | yes / no | yes / yes | yes / yes | no / no | no / no | yes / yes | yes | no / no | yes / yes | no | no |
| Patient 21 | 1 / 2 | 2 / 2 | M | 53 | 1 | no / yes | yes / yes | no / no | no / no | no / no | no / no | yes | no / no | no / yes | yes | MMF |
| Patient 22 | 3 / 3 | 3 / 3 | F | 51 | 1 | no / no | yes / yes | no / no | no / no | no / no | yes / yes | no | no / no | no / no | no | no |
| Patient 23 | 2 / 2 | 2 / 2 | F | 58 | 6 | yes / yes | yes / no | yes / yes | no / no | no / no | yes / yes | no | no / no | yes / no | yes | ciclosporin |
| Patient 24 | 2 / 2 | 2 / 2 | F | 51 | 18 | no / no | no / yes | no / no | no / no | no / no | yes / yes | no | no / yes | no / no | no | no |
| Patient 25 | 3 / 3 | 3 / 3 | F | 59 | 0 | yes / yes | no / no | no / no | yes / yes | no / no | no / no | no | no / yes | yes / yes | no | no |
| Patient 26 | 3 / 3 | 3 / 3 | F | 40 | 20 | yes / no | no / no | no / no | yes / yes | no / no | yes / yes | no | no / no | no / no | yes | MMF then stop |
| Patient 3 | 3 / 3 | 3 / 3 | F | 72 | 0 | no / no | yes / no | yes / no | no / no | no / no | no / yes | no | yes / no | no / no | no | no |
| Patient 4 | 0 / 0 | 2 / 2 | F | 53 | 0 | yes / yes | yes / yes | yes / yes | no / no | yes / no | yes / yes | yes | yes / no | yes / yes | yes | AZT then ADM |
| Patient 5 | 0 / 0 | 2 / 3 | F | 48 | 1 | yes / yes | yes / yes | yes / no | no / no | no / no | yes / yes | yes | yes / no | no / no | yes | AZT then MMF |
| Patient 6 | 0 / 0 | 2 / 2 | F | 32 | 0 | yes / yes | no / no | no / no | no / no | no / no | yes / yes | yes | yes / no | no / no | yes | MMF |
| Patient 7 | 2 / 2 | 2 / 2 | F | 52 | 2 | yes / yes | no / no | yes / yes | no / no | no / no | yes / yes | yes | yes / yes | no / no | yes | MMF |
| Patient 8 | 1 / 1 | 2 / 2 | F | 55 | 1 | yes / yes | no / no | yes / yes | no / no | no / no | no / no | yes | no / yes | no / no | yes | MMF then MMF + ADM |
| Patient 9 | 2 / 2 | 2 / 2 | F | 82 | 7 | no / no | no / yes | no / no | no / no | no / no | no / no | no | no / yes | no / yes | no | no |

**Supplemental Table 1 – Depigmentation grade, disease activity, therapeutic history per patient.**

Values shown as “OD / OS” for eye-specific fields. Kruijt grade refers to choroidal transparency/depigmentation (0–3) at the first and last visit. Histories (ODE, ME, venous vasculitis, CNV, anterior chamber cells/Tyndall, and vitreous cells/Hyalitis) indicate presence (yes) or absence (no) per eye. Corticosteroid history indicates exposure to systemic, sub-Tenon, or intravitreal steroids (per eye where applicable). Immunosuppression (yes/no) denotes systemic steroid-sparing therapy at any time; the adjacent column lists the agent(s) and sequence (e.g., MMF, adalimumab, azathioprine, cyclosporine, methotrexate), including treatment discontinuation when applicable.

Abbreviations: MO: Macular oedema; ODO: Optic disc oedema; CS: corticosteroids; IS: Immunosuppression; F: Female; M: Male; MMF, mycophenolate mofetil; MTX, methotrexate; ADM: adalimumab; AZT: azathioprine
