# Supplementary material for: Pigment loss and pseudo-albinism in Birdshot chorioretinitis
Source: Eye (Lond). 2026 Mar 5;40(8):1162–8. doi: 10.1038/s41433-026-04335-1 (PMC13195155; doi:10.1038/s41433-026-04335-1)
Supplement: Supplementary file 2 — Supplementary Table 2 [file 41433_2026_4335_MOESM2_ESM.docx]

|  |  | Estimate | SE | p-value |
| --- | --- | --- | --- | --- |
| BCVA (LogMAR) | **Kruijt Group 3** | 0.16 | 0.18 | 0.38 |
|  | **Time from First Symptoms to**  **Evaluation (Years)** | 0.01 | 0.03 | 0.66 |
|  | **History of MO** | 0.04 | 0.08 | 0.63 |
|  | **History of ODO** | 0.21 | 0.10 | **0.028** |
|  | **History of CNV** | 0.98 | 0.34 | **0.004** |
| MD (dB) | **Kruijt Group 3** | -0.73 | 1.78 | 0.68 |
|  | **Time from First Symptoms to**  **Evaluation (Years)** | -0.80 | 0.45 | 0.077 |
|  | **History of MO** | -1.42 | 1.12 | 0.20 |
|  | **History of CNV** | -3.35 | 2.08 | 0.11 |
| PSD (dB) | **Kruijt Group 3** | 0.42 | 0.45 | 0.35 |
|  | **Time from First Symptoms to**  **Evaluation (Years)** | 0.39 | 0.17 | **0.027** |
|  | **History of MO** | 0.64 | 0.48 | 0.19 |

**Supplementary Table 2. Multivariate GEE** **analysis of factors associated with functional outcomes (BCVA, MD, PSD)** **among the 26 included patients.**

Estimates are regression coefficients from exchangeable‑correlation Generalized Estimating Equations (GEE) models, with standard error (SE) and p‑value for each predictor. Time from First Symptoms is scaled per 5‑year increase. Kruijt group compares patients with Kruijt score 3 vs <3.

A**bbreviations:** BCVA: Best Corrected Visual Acuity (LogMAR), MD: Mean Deviation (dB), PSD: Pattern Standard Deviation (dB), SE: Standard Error, MO: Macular oedema, ODO: Optic disc oedema, CNV: Choroidal Neovascularization.
