# Supplementary material for: Pigment loss and pseudo-albinism in Birdshot chorioretinitis
Source: Eye (Lond). 2026 Mar 5;40(8):1162–8. doi: 10.1038/s41433-026-04335-1 (PMC13195155; doi:10.1038/s41433-026-04335-1)
Supplement: Supplementary file 3 — Supplementary Table 3 [file 41433_2026_4335_MOESM3_ESM.docx]

|  |  | Mean ± SD | | | p-value | | | |
| --- | --- | --- | --- | --- | --- | --- | --- | --- |
|  | **Interval**  **(years)** | **Kruijt 2** | **Kruijt 3** | **Literature** | **Trend**  **Kruijt 2** | **Trend**  **Kruijt 3** | **ABC**  **Kruijt 2** | **ABC**  **Kruijt 3** |
| BCVA | **0–5** | 0.08 ± 0.17 (n = 17) | 0.21 ± 0.21 (n = 12) | 0.09 ± 0.17 (n = 469) | **0.026** | **0.013** | 0.99 | **0.001** |
|  | **6–10** | 0.20 ± 0.24 (n = 21) | 0.40 ± 0.66 (n = 15) | 0.14 ± 0.28 (n = 499) |  |  |  |  |
|  | **11–15** | 0.25 ± 0.30 (n = 18) | 0.52 ± 0.88 (n = 16) | 0.21 ± 0.46 (n = 392) |  |  |  |  |
|  | **16–20** | 0.21 ± 0.42 (n = 9) | 0.71 ± 0.85 (n = 12) | 0.27 ± 0.49 (n = 293) |  |  |  |  |
|  | **21–25** | - (n = 1) | 1.18 ± 0.94 (n = 10) | 0.41 ± 0.63 (n = 154) |  |  |  |  |
| MD | **0–5** | -7.54 ± 7.08 (n = 15) | -9.83 ± 4.88 (n = 10) | -5.00 ± 4.70 (n = 466) | 0.058 | 0.99 | 0.99 | **0.007** |
|  | **6–10** | -6.73 ± 4.22 (n = 19) | -11.20 ± 8.38 (n = 13) | -5.40 ± 6.20 (n = 488) |  |  |  |  |
|  | **11–15** | -8.10 ± 4.52 (n = 17) | -12.52 ± 9.59 (n = 16) | -7.10 ± 8.70 (n = 392) |  |  |  |  |
|  | **16–20** | -6.55 ± 7.02 (n = 9) | -13.08 ± 9.43 (n = 12) | -7.70 ± 8.80 (n = 290) |  |  |  |  |
| PSD | **0–5** | 5.11 ± 3.66 (n = 15) | 4.64 ± 1.35 (n = 10) | 3.60 ± 1.90 (n = 466) | 0.30 | 0.42 | 0.11 | 0.90 |
|  | **6–10** | 5.04 ± 2.67 (n = 19) | 5.35 ± 1.58 (n = 13) | 4.10 ± 2.30 (n = 480) |  |  |  |  |
|  | **11–15** | 5.60 ± 2.68 (n = 17) | 5.80 ± 2.24 (n = 13) | 4.30 ± 2.60 (n = 392) |  |  |  |  |
|  | **16–20** | 5.21 ± 1.96 (n = 9) | 6.32 ± 2.68 (n = 10) | 4.70 ± 2.60 (n = 270) |  |  |  |  |

**Supplementary Table 3 – Evolution of visual function across time intervals compared to previously published data from the CO-BIRD cohort**

Display, for Kruijt depigmentation grades 2 and 3, of the observed mean ± SD (n eyes) of BCVA (logMAR), MD (dB) and PSD (dB) within each five‑year interval alongside published reference values; it also includes p‑values for the difference in longitudinal slope versus the literature trend from the CO-BIRD cohort (“Trend p”) and for the overall trajectory divergence via the area‑between‑curves permutation test (“ABC p”). Abbreviations: BCVA = best‑corrected visual acuity; MD = mean deviation; PSD = pattern standard deviation; CNV = choroidal neovascularization.
