# Supplementary figures and images for: Pigment loss and pseudo-albinism in Birdshot chorioretinitis
Source: Eye (Lond). 2026 Mar 5;40(8):1162–8. doi: 10.1038/s41433-026-04335-1 (PMC13195155; doi:10.1038/s41433-026-04335-1)

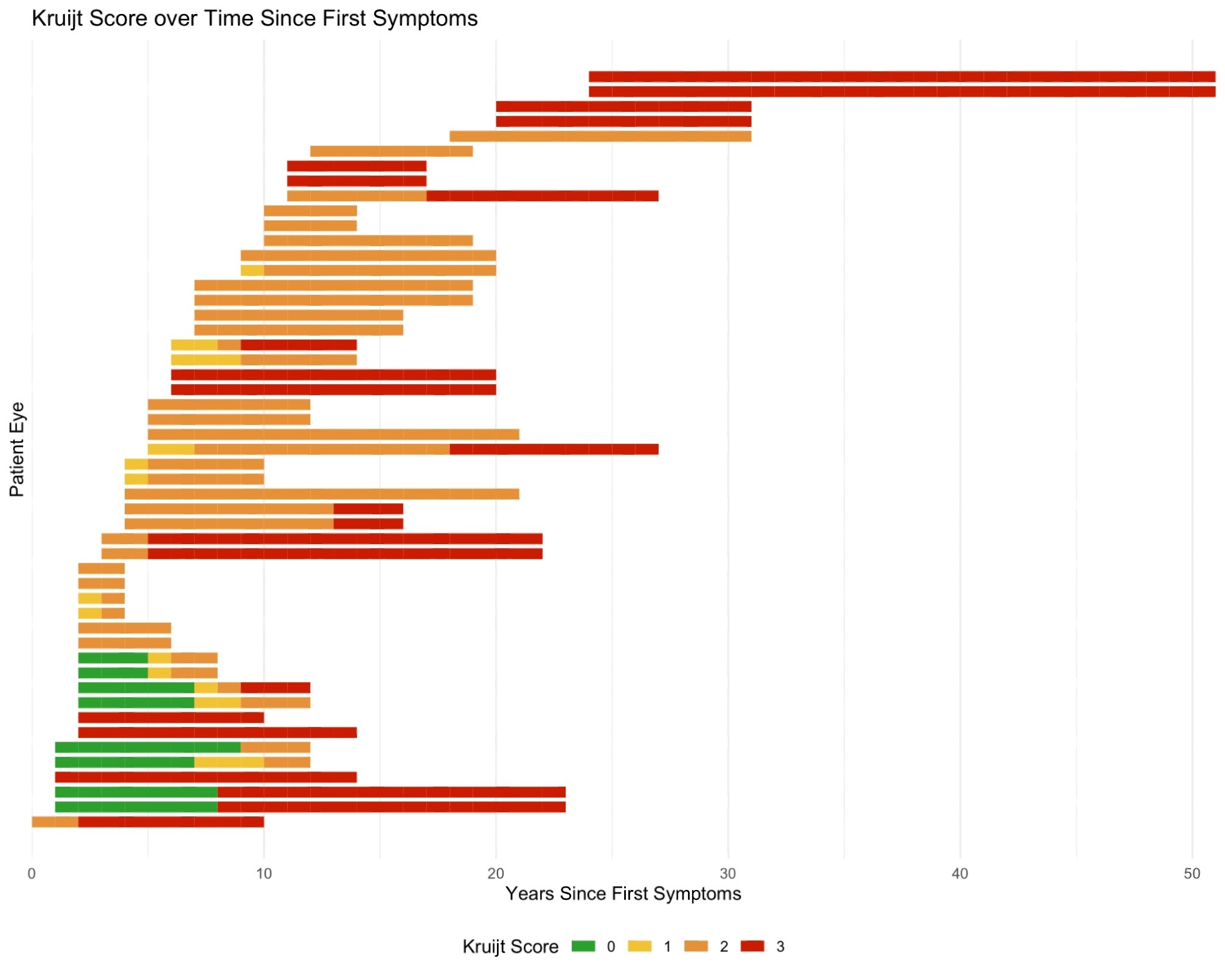

Supplement: Supplementary file 4 — Supplementary Figure 1 [file 41433_2026_4335_MOESM4_ESM.jpg]

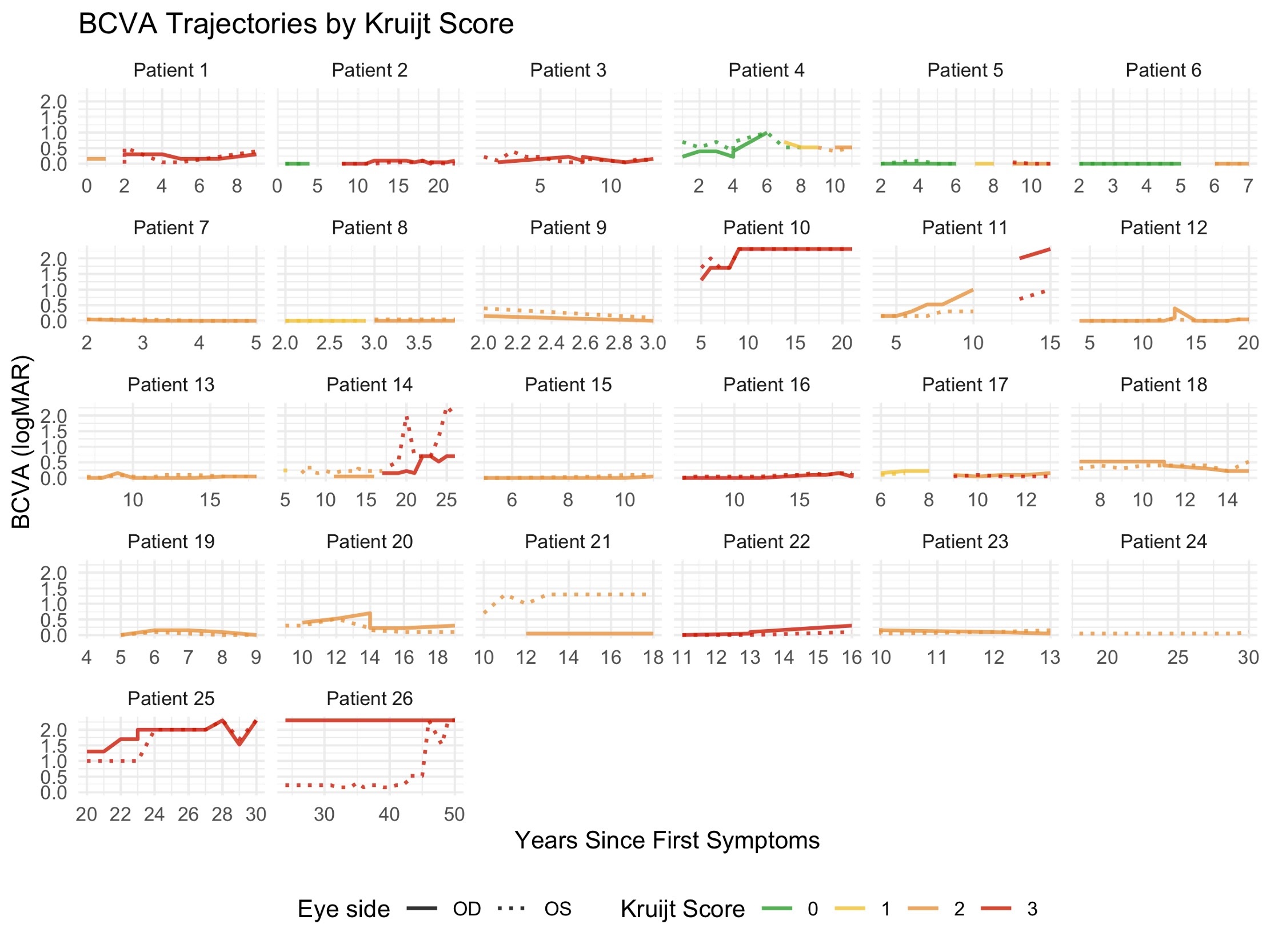

Supplement: Supplementary file 5 — Supplementary Figure 2 [file 41433_2026_4335_MOESM5_ESM.jpg]

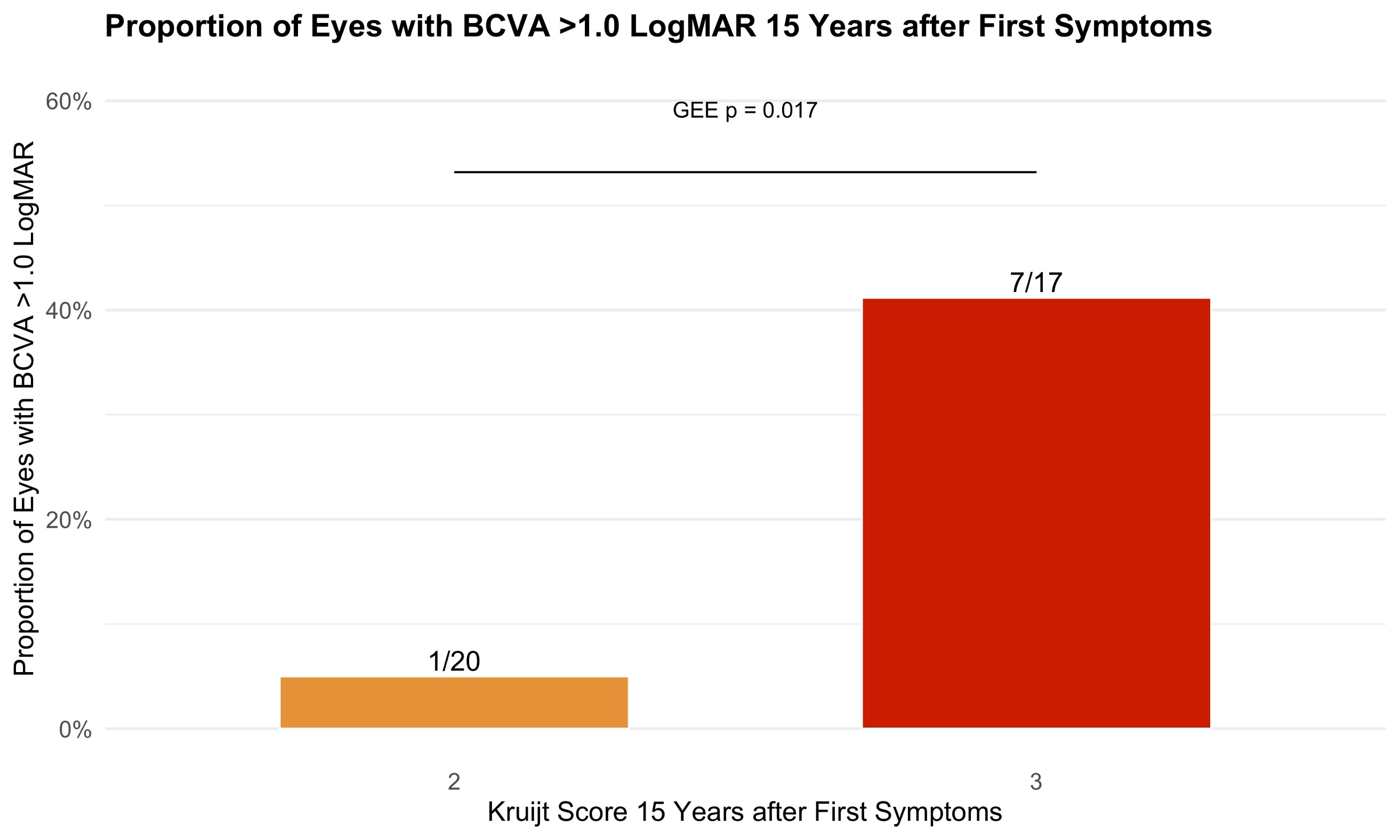

Supplement: Supplementary file 6 — Supplementary Figure 3 [file 41433_2026_4335_MOESM6_ESM.jpg]

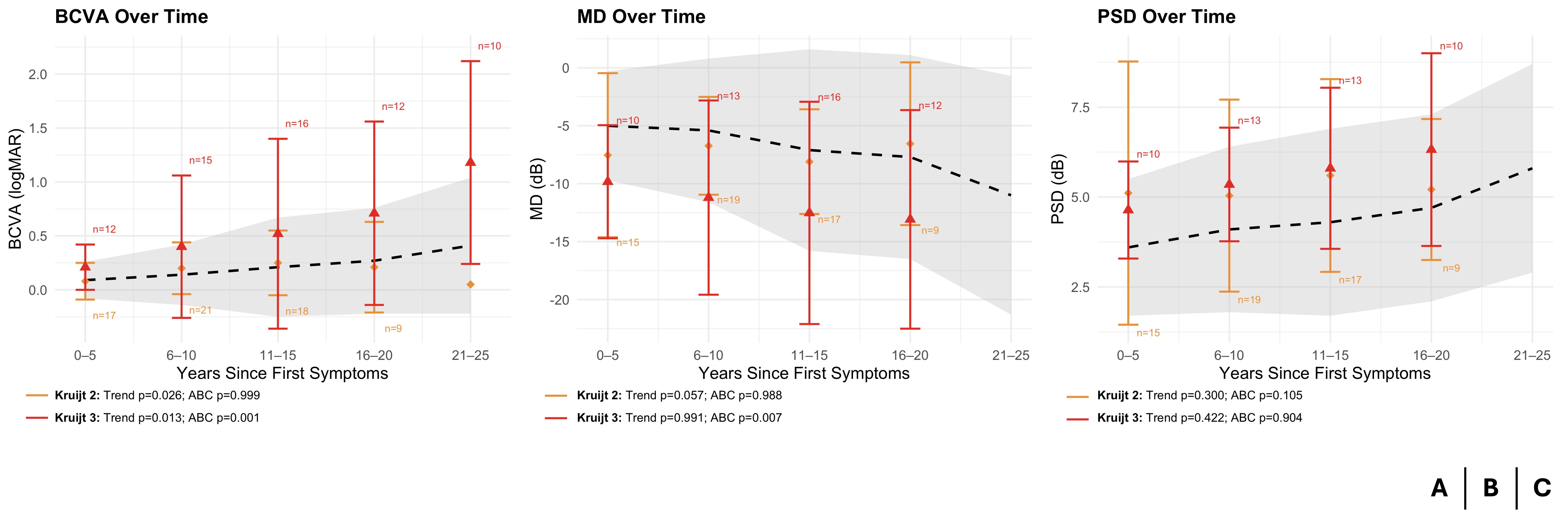

Supplement: Supplementary file 7 — Supplementary Figure 4 [file 41433_2026_4335_MOESM7_ESM.jpg]
